# Inhibition of Glial Activation and Subsequent Reduction in White Matter Damage through Supplementation with a Combined Extract of Wheat Bran, Citrus Peel, and Jujube in a Rat Model of Vascular Dementia

**DOI:** 10.3390/cimb46020096

**Published:** 2024-02-11

**Authors:** Ki Hong Kim, Sun-Ha Lim, Jeong Hyun Hwang, Jongwon Lee

**Affiliations:** 1 Department of Neurosurgery, School of Medicine, Daegu Catholic University, Daegu 42105, Republic of Korea; gneuros@cu.ac.kr; 2 DigmBio, Inc., Seongnam 13486, Republic of Korea; sunha1977@hanmail.net; 3 Department of Neurosurgery, School of Medicine, Kyungpook National University, Daegu 41944, Republic of Korea; jhwang@knu.ac.kr; 4 Department of Biochemistry, School of Medicine, Daegu Catholic University, Daegu 42105, Republic of Korea

**Keywords:** wheat, citrus, jujube, phytic acid, vascular dementia, chronic hypoperfusion, white matter

## Abstract

Vascular dementia (VaD) is the second most common type of dementia after Alzheimer’s disease. In our previous studies, we showed that wheat bran extract (WBE) reduced white matter damage in a rat VaD model and improved memory in a human clinical trial. However, starch gelatinization made the large-scale preparation of WBE difficult. To simplify the manufacturing process and increase efficacy, we attempted to find a decoction containing an optimum ratio of wheat bran, sliced citrus peel, and sliced jujube (WCJ). To find an optimal ratio, the cell survival of C6 (rat glioma) cultured under hypoxic conditions (1% O_2_) was measured, and apoptosis was assessed. To confirm the efficacies of the optimized WCJ for VaD, pupillary light reflex, white matter damage, and the activation of astrocytes and microglia were assessed in a rat model of bilateral common carotid artery occlusion (BCCAO) causing chronic hypoperfusion. Using a combination of both searching the literature and cell survival experiments, we chose 6:2:1 as the optimal ratio of wheat bran to sliced citrus peel to sliced jujube to prepare WCJ. We showed that phytic acid contained only in wheat bran can be used as an indicator component for the quality control of WCJ. We observed in vitro that the WCJ treatment improved cell survival by reducing apoptosis through an increase in the Bcl-2/Bax ratio. In the BCCAO experiments, the WCJ-supplemented diet prevented astrocytic and microglial activation, mitigated myelin damage in the corpus callosum and optic tract, and, consequently, improved pupillary light reflex at dosages over 100 mg/kg/day. The results suggest that the consumption of WCJ can prevent VaD by reducing white matter damage, and WCJ can be developed as a safe, herbal medicine to prevent VaD.

## 1. Introduction

Vascular dementia (VaD) accounts for 15–20% of all dementia and is the second most common form of dementia after Alzheimer’s disease (60–70%) [1]. VaD is caused when large and small cerebral arteries are partially or completely blocked, and the brain then suffers from an inadequate blood supply, which can ultimately lead to cognitive impairment [2,3]. In hypoperfusion dementia, large arteries, such as the common carotid arteries (CCAs), are occluded [4,5,6]. This results in an insufficient blood supply to vulnerable regions like subcortical white matter and ultimately leads to the activation of microglia and astrocytes; subsequently, damage occurs to white matter, including demyelination and axonal damage [4,5]. To study VaD in rats, the bilateral common carotid artery occlusion (BCCAO) model is most commonly used because the model entails a white matter lesion [2,7]. In the rat BCCAO model, white matter damage occurs in the corpus callosum (CC), optic tract (OPT), and internal capsule (IC) [7,8], of which an injury in the optic nerve impairs the pupillary light reflex (PLR), controlling the pupil diameter in response to light intensity [9]. Furthermore, astrocytic and microglial activation also occur in the rat BCCAO model [10]. In summary, the rat BCCAO model is appropriate for studying human VaD since this model accompanies the activation of astrocytes and microglia, as well as the disintegration of myelin.

In our previous studies, we have shown that the supplementation of wheat bran extract (WBE) reduced white matter damage in the rat BCCAO model [11]. Moreover, in a randomized controlled trial, the intake of WBE improved vision-related memory, such as visual memory and visuospatial working memory, in cognitively normal, elderly adults with subjective memory impairments [12]. As vision-related cognition also involves white matter, the findings from these animal and human studies suggest that WBE intake might be effective in the prevention of VaD. However, scaling up WBE manufacturing for commercial development is a challenging process due to the fact that it is difficult to remove starch, which is not an active ingredient, from the extract. When hot water is used for extraction, the wheat starch gelatinizes at 60 °C and makes its removal from the extract difficult. An alternative is to use a combination of cold water extraction and decanter centrifugation [11], which is very complex and expensive. In this study, we attempted to use another method that involves a much simpler process; when wheat bran is mixed with other plants and extracted using hot water, the gelatinized starch binds to the residue and can be easily removed without centrifugation. We examined various decoctions of wheat and a combination of other plants to find out which mix retained or potentially surpassed plain WBE extract’s efficacy related to VaD.

After conducting a search of the literature, we decided on using the Gan Mai Da zao decoction (herbal medicine in Chinese) as a candidate. The Gan Mai Da zao decoction consists of *Triticum aestivum* L. (common wheat), *Glycyrrhiza uralensis* Fisch. (licorice), and *Ziziphus jujube* Mill. (jujube). This decoction was selected for its inclusion of wheat as a constituent and because of its efficacy against such neuropsychiatric diseases, such as depression and post-traumatic stress disorder, in both animal models [13] and humans [14]. Unfortunately, there are some side effects to the long-term consumption of licorice, a necessary ingredient of the Gan Mai Da zao decoction, including hypertension and hypokalemic-induced secondary complications [15]. Therefore, we replaced licorice with *Citrus unshiu* (citrus) peel, which is deemed safe [16] and is also associated with a reduced risk of incident dementia in humans [17]. Citrus peel also reduced brain injury in a rat model of ischemic stroke, which is also a cause of VaD [18]. Based on these findings, we hypothesized that the extract of wheat bran, citrus peel, and jujube (WCJ), prepared by the standard production procedure of boiling the mixture in water, filtering it, condensing it, and drying the condensate, would reduce white matter damage in the rat BCCAO model due to each ingredient’s efficacy in reducing brain injury under ischemic conditions [11,18,19].

To test this hypothesis, we first determined the optimal ratio of the ingredients for maximum efficacy. In preparing the decoctions, we used response surface methodology to determine the optimal extraction conditions [20]. To reduce the number of experiments required to find the optimal ratio of the three ingredients, we started with the optimal ratio between wheat and jujube found in the literature [13]. Then, we determined the optimal ratio between citrus peel and jujube using a C6 cell culture under hypoxic conditions to mimic the conditions in the BCCAO model [10]. C6 cells are a good proxy for neurons in the brain because they exhibit the oligodendrocyte phenotype that produces myelin in the brain [21]. Cell survival, defined in this study as the percentage of live cells compared to the control, and apoptotic cascades were assessed using C6 cells treated with the optimized WCJ extract. For the quality control of WCJ, we chose phytic acid as an indicator component because wheat bran is the major ingredient required to prepare WCJ, and phytic acid is mainly found in grains, including wheat [22]. Finally, we investigated whether supplementation with the optimized WCJ extract could reduce brain damage in the rat BCCAO model, in which bilateral CCAs were occluded for 30 days [23].

## 2. Materials and Methods

### 2.1. Preparation of WCJ

Wheat bran, a milling by-product of a domestic variety, was purchased from a cultivator in the Republic of Korea [23]. Wheat bran was sieved with a 500-μm mesh (Chung Gye Sang Gong Sa, Seoul, Republic of Korea) to remove starch granules. Sliced citrus peel and sliced jujube, both cultivated in Korea and prepared in accordance with Good Manufacturing Practices, were purchased from Hanirang (Yeongcheon, Gyeongsangbuk-do, Republic Korea). To prepare the decoctions, each ingredient, separately or as a mixture, was boiled in an extractor for 1 h in 10-fold excess water (Daewoong DWP-2000, Seoul, Republic of Korea). The hot extract was then filtered through a 125- or 250-μm mesh (Chung Gye Sang Gong Sa, Seoul, Republic of Korea). The hot filtrate was concentrated using a vacuum evaporator (N-1000, EyelA Co., Tokyo, Japan). The resulting concentrate was air-dried at 60 °C and pulverized into powder.

### 2.2. Measurement of Phytic Acid Contents

The contents of phytic acid in each ingredient and WCJ were measured using a colorimetric assay developed by Megazyme International Ireland (County Wicklow, Ireland) [24]. Briefly, each raw ingredient, the extract of each ingredient, and the WCJ (1 g, respectively) were vigorously mixed in hydrochloric acid (0.66 M, 20 mL). The extract (1 mL) was centrifuged, and the supernatant (0.5 mL) was neutralized with sodium hydroxide (0.75 M, 0.5 mL). Inorganic phosphate was released from phytic acid with phytase and alkaline phosphatase. The inorganic phosphate that was released was reacted with ammonium molybdate to produce molybdenum blue. The amount of inorganic phosphate was assessed after absorbance was measured at 655 nm.

### 2.3. Cell Culture

The cell culture for *Rattus norvegicus* glioma cells (C6), purchased from the Korean Cell Line Bank (Seoul, Republic of Korea), was performed as described previously [18]. The cells were plated in a 2 × 10^5^ cells/12-well plate culture dish and grown in Dulbecco’s modified Eagle’s medium (DMEM) supplemented with 10% fetal bovine serum (FBS, Gibco BRL, Gaithersburg, MD, USA), 100 units/mL of penicillin, and 100 μg/mL of streptomycin (Gibco BRL). They were kept at 37 °C for 1 day under normoxic conditions (5% CO_2_; balanced with air) in a humidified chamber (Forma Scientific, Inc., Marietta, OH, USA).

Within these experiments, to determine the optimal WCJ ratio, the culture medium under normoxic conditions was exchanged with a fresh medium containing various combinations of sliced jujube extract and sliced citrus peel extract at various concentrations (100, 200, 300, and 400 μg/mL). After 24 h of cell culture under hypoxic conditions (1% O_2_, 5% CO_2_, and balanced with N_2_), viable cells were stained with a 0.4% trypan blue dye solution and counted using a hemocytometer. In the experiments assessing the WCJ dosage for maximal cell survival, the culture medium under normoxic conditions was exchanged with a fresh medium containing 200, 400, or 800 μg/mL of WCJ, pre-dissolved in 10% ethanol, or that containing no WCJ (control). After 0, 6, 12, 24, and 30 h of cell culture under hypoxic conditions (1% O_2_, 5% CO_2_, and balanced with N_2_) in a humidified chamber (ASTEC Co., Ltd., Tokyo, Japan), viable cells were stained with a 0.4% trypan blue dye solution and counted using a hemocytometer.

C6 cells (1 × 10^5^ cells/600 μL in a 4-chamber slide (Thermo Fisher Scientific Inc., Rochester, NY, USA)) were used for terminal deoxynucleotidyl transferase-mediated dUTP nick end-labeling (TUNEL) staining. The cells (1 × 10^6^ cells/60 mm dish) cultured under the same culture conditions as those for the TUNEL staining were used for the Western blotting experiments.

### 2.4. Terminal Deoxynucleotidyl Transferase-Mediated dUTP Nick End-Labeling (TUNEL) Staining

To investigate whether the WCJ treatment improved cell survival through the inhibition of apoptosis, we used the TUNEL assay to measure the DNA nicks generated in the cells as a biomarker for apoptotic cells [25]. TUNEL staining was performed as described previously [26,27]. C6 cells were fixed in a 10% neutral-buffered formalin solution at room temperature for 30 min. Endogenous peroxidase was inactivated by the treatment with 0.3% hydrogen peroxide in methanol for 30 min at room temperature. The cells were further incubated in a permeabilizing solution (0.1% sodium citrate and 0.1% Triton X-100) for 2 min at 4 °C. Then, the TUNEL reaction mixture was added to the cells for 60 min at 37 °C, followed by labeling with a peroxidase-conjugated anti-goat antibody for an additional 30 min. After being stained with diaminobenzidine for 10 min and counterstained with methyl green for 5 min to identify all the nuclei, the cells were rinsed with phosphate-buffered saline (PBS), mounted with 50% glycerol, and examined under a microscope. Micrographs of TUNEL-positive nuclei and methyl green-stained nuclei were captured using an Olympus microscope (Hicksville, NY, USA). ImageJ software (NIH, v1.47) was used to count the number of cells in 10 random fields. The WCJ (800 μg/mL) and control groups represent cells at 24 h of cell culture with and without the WCJ treatment, respectively, under hypoxic conditions. For quantitative analysis, ratios (apoptotic cells/total cells) were presented.

### 2.5. Western Blotting

To investigate a biomarker located upstream of the DNA nicks, the levels of anti-apoptotic Bcl-2 and pro-apoptotic Bax were measured using Western blotting [28]. Western blotting for Bcl-2 and Bax was performed as described previously [27]. Proteins were extracted from C6 cells cultured as described in the “cell culture” section above using RIPA buffer (Cell Signaling Technology, Beverly, MA, USA) that contained a cocktail of protease and phosphatase inhibitors (Roche, Mannheim, Germany). The protein extracts were separated into sodium dodecyl sulfate (SDS)–polyacrylamide gels. The separated proteins were transferred to polyvinylidene fluoride membranes (Millipore, Darmstadt, Germany). The membranes were blocked at room temperature with 5% non-fat skim milk for 1 h and incubated overnight with the corresponding primary antibodies at 4 °C with gentle shaking. The primary antibodies for Bcl-2 and Bax were purchased from Santa Cruz Biotechnology, Inc. (1:1000, Santa Cruz, CA, USA). Horseradish peroxidase-labeled secondary antibodies (1:2000, Enzo, Farmingdale, NY, USA) were used. The membranes were developed with Amersham ECL Prime Western Blotting Detection Reagent (GE Healthcare, Little Chalofont, UK) using the ChemiDoc XRS Gel Imager (Bio-Rad Laboratories, Inc., Hercules, CA, USA). Protein bands were quantified using Image Lab Software (Bio-Rad Laboratories, v5.1, Hercules, CA, USA). β-Actin was used as a loading control. The relative band intensity values for Bcl-2 and Bax were calibrated by setting the value of the sham group as 1. The sham, control, and WCJ (800 μg/mL) groups represent cells harvested at 0 h of cell culture without WCJ treatment, 24 h of cell culture without WCJ treatment, and 24 h of cell culture with WCJ treatment, respectively, under hypoxic conditions.

### 2.6. Animals

Eight-week-old male Sprague Dawley rats were purchased from Samtaco Inc. (Osan, Gyeonggi-do, Republic of Korea). Experiments were carried out according to the guidelines for animal care and protocols for laboratory animal use approved by the Institutional Animal Care and Research Advisory Committee of Daegu Catholic University, Daegu, Republic of Korea (approval number: DCIAFCR-170208-3-Y). The rats were housed in a temperature-controlled environment with diet and water available ad libitum under diurnal lighting conditions until the start of the experiments.

### 2.7. Diet Preparation

A diet containing WCJ was prepared using a semisynthetic diet as previously described [23,27]. To prepare 1 kg of 100 mg/kg and 400 mg/kg WCJ diets, mixtures of 2 g WCJ and 48 g corn starch and 8 g WCJ and 42 g corn starch, respectively, were added to 950 g of a modified AIN-93G diet purchased from Unifaith Inc. (Seoul, Republic of Korea). For 1 kg of basal diet, 50 g of corn starch was added to 950 g of the modified AIN-93G diet.

### 2.8. Diet Administration

**Figure 1:**
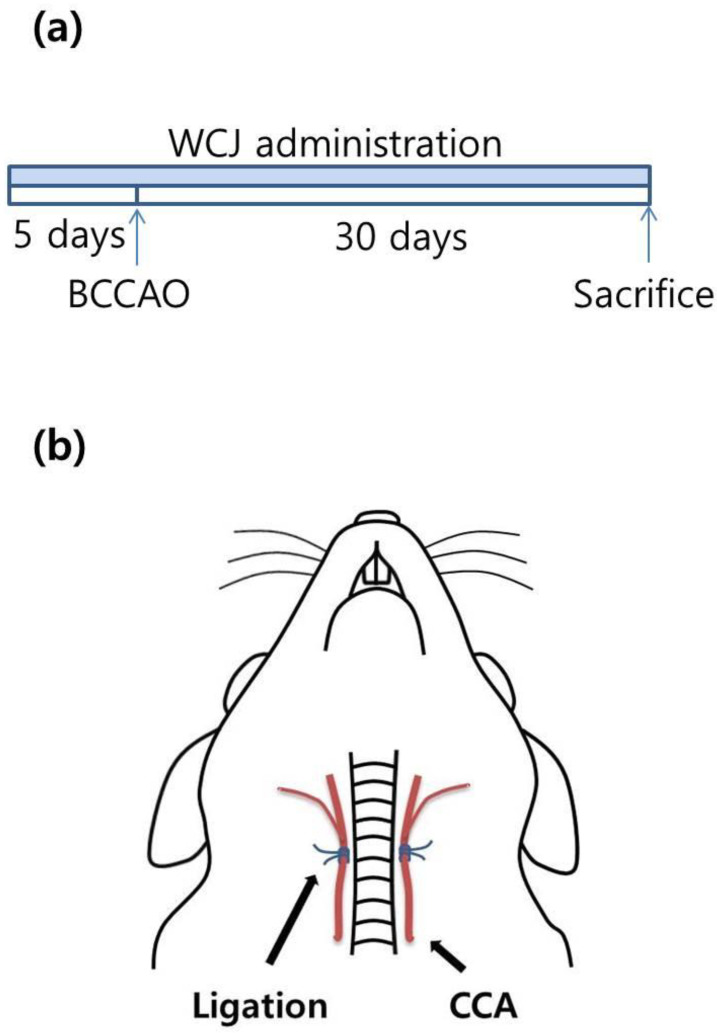
Experimental schemes. (**a**) WCJ (wheat bran, citrus, and jujube) was administered for 5 days before and 30 days after the rats underwent BCCAO (bilateral common carotid artery occlusion) surgery. (**b**) The CCAs (bilateral common carotid arteries) in the control and WCJ-treated groups were exposed and ligated permanently with silk sutures, leaving the brain under chronic hypoperfusion.

WCJ and basal diets were supplied to the rats as described previously [11,27]. The rats were randomly assigned to one of four groups, namely sham (*n* = 6), control (*n* = 6), and WCJ-treated (100 and 400 mg/kg/day) (*n* = 6, and 6). Six rats were used in each group; this number was enough to obtain reliable results when WBE was used in the same rat BCCAO model in our previous study [11]. In the WCJ-treated group, the rats (approximately 300 g) received 15 g/day of a 100 and 400 mg/kg WCJ diet, respectively, for 5 days before and 30 days after the rats underwent BCCAO surgery (Figure 1a). Every day, once the rats consumed all the WCJ diet, more basal diet was provided ad libitum. In the control and sham groups, the rats were supplemented only with the basal diet.

### 2.9. Surgical Procedures for BCCAO

A bilateral CCA ligation procedure was performed on the rats, as described previously [11,23]. Throughout the entire surgical procedure, the rats were under a 20%/80% oxygen/nitrous oxide condition and anesthetized using isoflurane inhalation (Hana Pharmaceutical Inc., Seoul, Republic of Korea). In the WCJ-treated and control groups, a midline incision exposed the bilateral CCAs, which were then ligated with 4-0 silk sutures (Figure 1b). In the sham group, the rats received the same procedure except for the ligation. During surgery, the rectal temperature was maintained at 37 ± 0.5 °C with a thermostatically controlled warming plate (Harvard Apparatus, Holliston, MA, USA). Thirty days after BCCAO, the rats were assessed for their PLR. The rats were then sacrificed to yield brains, which were harvested for further study. Throughout the BCCAO experiments, none of the Sprague Dawley rats died. This observation is consistent with a report showing that Sprague Dawley rats have a much lower mortality rate than Wistar rats in a rat BCCAO model, which is similar to the model used in this study (mortality rate: 1/15 vs. 4/15 rats) [29].

### 2.10. Assessment of the Pupillary Light Reflex (PLR)

To assess whether a reduction in white matter damage manifests itself as a change in any bodily function, we used the PLR as an indicator. The PLR was assessed as described previously [11,23]. Before BCCAO surgery, we examined the PLR of each rat and selected rats with normal PLR function to undergo surgery. Before the rats were sacrificed, each rat was adapted to darkness for at least 5 min. One eye was exposed to a beam of light from a clinical brand (3M Korea, Ltd., Seoul, Republic of Korea). The rat was then allowed to readapt to darkness for approximately 1 min, after which the PLR of the other eye was assessed. A loss of the PLR was defined as the failure of the pupil to constrict after 10 s of exposure to light [11]. After the PLR experiment, the rats in each group were divided into 2 groups: abnormal rats that lost the PLR in one or both eyes and normal rats that maintained their PLR in both eyes.

### 2.11. Luxol Fast Blue Staining

To assess white matter damage, Luxol fast blue staining was performed on the sections of the rat brains harvested. Luxol fast blue staining was performed as described previously [11,23]. The harvested brain was cut into slices, namely into the CC, IC, and OPT regions. Then, the slices were fixed in formalin and embedded in paraffin, from which 5-μm-thick sections were prepared for Luxol fast blue staining. The severity of damage in the three regions was assessed by an examiner, who was blinded to the experimental conditions and graded the samples as being characterized by normalcy (Grade 0), disarrangement of nerve fibers (Grade 1), formation of marked vacuoles (Grade 2), and disappearance of myelinated fibers (Grade 3) [30].

### 2.12. Immunohistochemical Staining

Astrocytes were activated, accompanied by hypertrophy and proliferation of the astrocytes, which can be detected with immunohistochemical staining against GFAP [10]. Microglia were also activated, accompanying the morphological transformation and proliferation of the microglia. The activation of microglia can be detected with immunohistochemical staining against ionized calcium-binding adaptor molecule 1 (Iba1) [23]. The induction of astrocytic and microglial activation was responsible for neuroinflammation and, subsequently, white matter damage in the rat BCCAO model [31,32]. Immunohistochemical staining was performed as described previously [11,23]. The prepared sections were treated by boiling in a microwave, submerging in hydrogen peroxide (0.03%) in methyl alcohol, and incubating in 1% bovine serum albumin and 5% normal serum. Then, the sections were incubated with primary antibodies against glial fibrillary acidic protein (GFAP, 1:100, BD PharMingen, San Diego, CA, USA) or ionized calcium-binding adaptor molecule 1 (Iba1, 1:200, Wako, Osaka, Japan). After the sections were washed, they were treated with a biotinylated secondary antibody (1:200, Vector Laboratories, Burlingame, CA, USA), incubated with an avidin–biotin peroxidase complex (Elite Vectastain ABC kit, Vector Laboratories, Burlingame, CA, USA), and visualized with diaminobenzidine (Roche, Mannheim, Germany). Immunostained sections were captured using an Olympus microscope (200×) (Olympus Corporation, Tokyo, Japan). Quantitative analysis was performed using ImageJ software (NIH, v1.47). The area covered by the GFAP-positive astrocytes and Iba1-positive microglia was computed as a percentage of the total area. To assess the GFAP and Iba1 levels in the WCJ-treated and control groups, the values were calibrated by setting the sham group’s value as 1.

### 2.13. Statistical Analyses

Values were expressed as means ± SEM. Statistical analyses were performed using SPSS software (IBM SPSS Statistics; version 19, Armonk, NY, USA). The Shapiro–Wilk test and Levene’s test were each used to test all the variables for the normal distribution and homogeneity of the variances, respectively. Comparisons among multiple groups were analyzed using a one-way ANOVA with Welch’s correction factor and post-hoc Dunnett’s T3. The comparison of the apoptosis extent was analyzed using the Student’s *t*-test. The results were considered significant at *p* < 0.05.

## 3. Results

### 3.1. Optimization of Ingredient Ratios to Prepare WCJ

**Figure 2:**
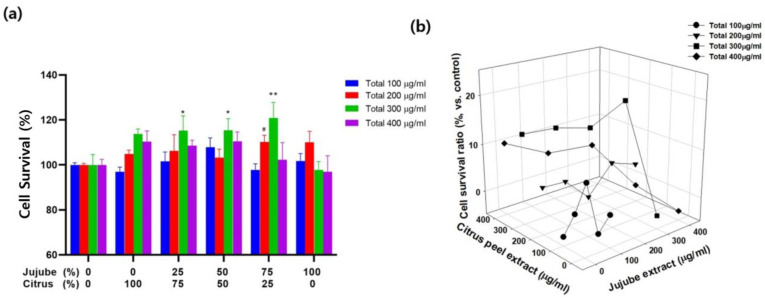
Determination of optimal ratios between citrus peel extract and jujube extract to prepare WCJ. (**a**) Cell survival (%) was assessed in the two dimensions after 24 h of cell culture under hypoxic conditions (1% O_2_, 5% CO_2_, and balanced with N_2_) using a 0.4% trypan blue dye solution at various combinations of citrus peel extract and jujube extract (100:0, 75:25, 50:50, 25:75, and 0:100) at various total concentrations (0, 100, 200, 300, and 400 μg/mL). Cell survival was compared at various combinations of citrus peel extract and jujube extract at the same total concentrations. ** *p* < 0.01 and * *p* < 0.05 for 300 μg/mL, and ^#^ *p* < 0.05 for 200 μg/mL vs. the control group (24 h of cell culture without WCJ treatment under hypoxic conditions). (**b**) Cell survival was assessed in the three dimensions for the same data used in (**a**). Numbers at the axes of “Citrus peel extract” and “Jujube peel extract” represent the concentrations of citrus peel extract and jujube peel extract added into the culture medium, respectively. Numbers at the axis of “Cell survival ratio (%)” represent an improvement in cell survival compared with the control group (24 h of cell culture without WCJ treatment under hypoxic conditions).

As common wheat and jujube are ingredients found in both WCJ and the Gan Mai Da zao decoction, we searched the literature and learned that 3:1 is the ratio between common wheat and jujube in the Gan Mai Da zao decoction [13]. In this study, we found that the extraction yields of wheat bran and sliced jujube were approximately 20% and 40%, respectively. Thus, in order to obtain a 3:1 ratio of wheat bran to jujube extracts in WCJ, we used 6:1 as our input ratio between the two ingredients, ignoring the fact that whole wheat instead of wheat bran has been used in the preparation of the Gan Mai Da zao decoction in general. Next, to determine the optimal ratio between citrus peel and jujube, we tested various combinations of sliced citrus peel extract and sliced jujube extract at various total concentrations (100, 200, 300, and 400 μg/mL) using C6 cell cultures at 24 h under hypoxic conditions (Figure 2a). Cell survival was significantly increased at the 0.25:0.75, 0.5:0.5 and 0.75:0.25 ratios for a 300 μg/mL sample compared with the control sample (24 h of cell culture without WCJ treatment under hypoxic conditions) (*p* < 0.01, 0.05, and 0.05, respectively) and the 0.25:0.75 ratio for a 200 μg/mL sample compared with the control sample (*p* < 0.05). To choose the appropriate ratios more easily, we showed the data in Figure 2a in the three dimensions to adopt response surface methodology without error bars (Figure 2b). Overall, cell survival was maximized at a 1:1 ratio for the 100 and 400 μg/mL samples, and at a 0.25:0.75 ratio for the 200 and 300 μg/mL samples. We chose a 1:1 ratio since sliced citrus peel is cheaper than sliced jujube by weight in the Republic of Korea. Combining this decision with our finding that the extraction yields of sliced citrus peel and sliced jujube were 20% and 40%, respectively, we decided on a 2:1 ratio between sliced citrus peel and sliced jujube to make a 1:1 ratio of extracted ingredients in WCJ. Finally, based on these results, we concluded that 6:2:1 is the optimal input ratio between wheat bran, sliced citrus peel, and sliced jujube in preparing the optimal WCJ extract.

### 3.2. Phytic Acid Content in WCJ

Phytic acid only exists significantly in wheat bran and wheat bran extract (3.5 and 5.2%, respectively) (Table 1). This result is consistent with a previous report, in which the content of phytic acid in wheat bran was in the range of 2.1~7.3% [22]. Therefore, phytic acid can be used as an indicator component representing WCJ. WCJ contains 2.2% phytic acid derived from wheat bran. Taken together, we can estimate the wheat bran used to prepare WCJ by comparing the contents of phytic acid in wheat bran and WCJ.

### 3.3. Improvement in Cell Survival through WCJ Treatment by Apoptosis Inhibition under Hypoxic Conditions

**Figure 3:**
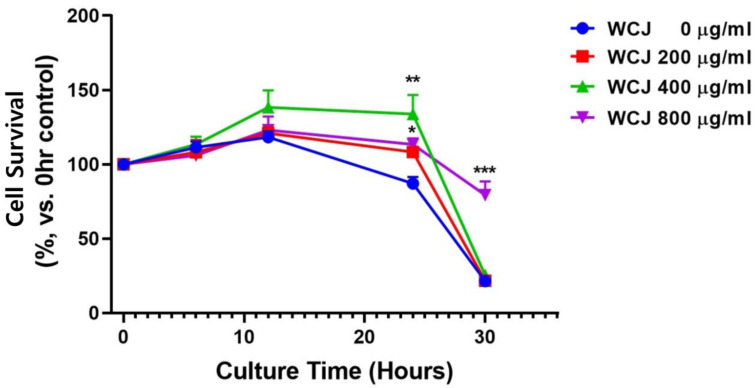
Assessment of C6 cell survival through WCJ treatment. The ratios for viable cells (cell survival) at various culture times were presented by setting the value at 100 for the viable cells harvested at 0 h of cell culture under hypoxic conditions. *** *p* < 0.001, ** *p* < 0.01, and * *p* < 0.05 vs. the control group (0 h of cell culture without WCJ treatment under hypoxic conditions).

**Figure 4:**
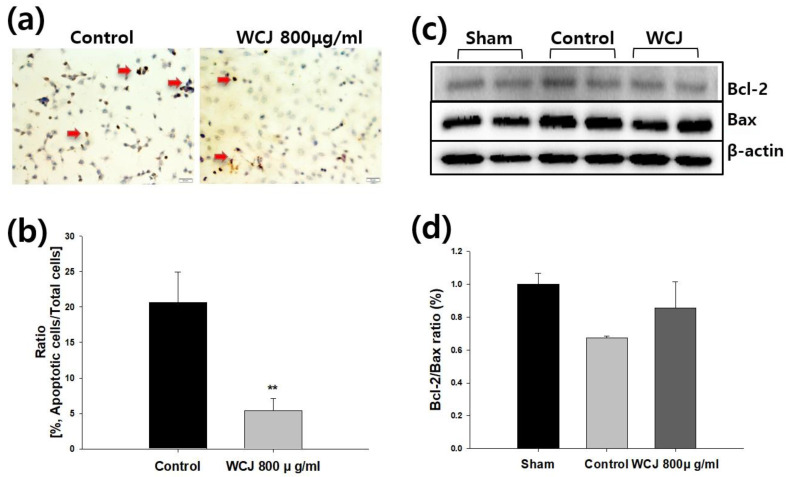
Assessment of apoptotic cascades for C6 cells through WCJ treatment. (**a**) The stained cells (red arrow) represent apoptotic cells (TUNEL staining, ×400). Scale bar, 50 µm. (**b**) The quantitative analysis of apoptotic cells is presented. Apoptotic cells were significantly reduced under the WCJ treatment. ** *p* < 0.01 vs. the control group. (**c**) Western blots for Bcl-2 and Bax are presented. β-actin was used as a loading control. (**d**) The quantitative analysis of the Bcl-2/Bax ratio is presented. The higher ratio of the WCJ-treated group represented an improvement in cell survival. ** *p* < 0.01 vs. the control group.

When C6 cells were cultured at various times under hypoxic conditions in the presence of WCJ (200, 400, and 800 μg/mL), WCJ treatment at 400 and 800 μg/mL significantly improved cell survival at 24 h of cell culture (*p* < 0.01 and *p* < 0.05, respectively), and WCJ treatment at 800 μg/mL improved cell survival significantly at 30 h of cell culture (*p* < 0.001) (Figure 3). We performed the TUNEL assay in the culture condition (800 μg/mL of WCJ and 24 h of cell culture), in which WCJ treatment significantly improved cell survival (Figure 4). Qualitatively, the WCJ treatment reduced the number of stained cells representing apoptotic cells compared with the control group (Figure 4a). Quantitatively, the WCJ treatment significantly reduced the number of apoptotic cells compared with the control group (*p* < 0.01) (Figure 4b). These findings show that the WCJ treatment inhibits the occurrence of apoptosis. A representative result of Western blotting is shown in Figure 4c. The Bcl-2/Bax ratio, which determines cell survival, tended to be higher in the WCJ-treated group compared with the control group (Figure 4d). These results suggest that the WCJ treatment might skew Bcl-2 and Bax expression in the direction of inhibiting apoptosis. Taken together, the WCJ treatment improved cell survival under hypoxic conditions by inhibiting apoptotic cascades.

### 3.4. Reduction in PLR Loss through WCJ Supplementation

In the sham group, all six (100%) rats showed PLRs in both eyes, indicating that they maintained normal PLRs without damage (Table 2). In the control group, however, five (83%) rats lost PLRs in one or both eyes, and only one (17%) rat retained PLRs in both eyes. In the WCJ-treated group supplemented with WCJ at a dosage of 100 mg/kg/day, the number of rats that lost PLRs in one or both eyes was reduced from five (83%) to three (50%), and the number of rats that maintained PLRs in both eyes was increased from one (17%) to three (50%) compared with the control group. Finally, in the WCJ-treated group supplemented with WCJ at a dosage of 400 mg/kg/day, the number of rats that lost PLRs in one or both eyes was reduced from five (83%) to one (17%), and the number of rats that maintained PLRs in both eyes was increased from one (17%) to five (83%) when compared with the control group. Differences between groups in terms of the loss of PLR were statistically significant (*p* = 0.015). These findings indicate that the increased supplementation of WCJ enhances functional recovery in PLR and is inversely correlated with white matter damage.

### 3.5. Reduction in White Matter Damage in the CC and OPT through WCJ Supplementation

**Figure 5:**
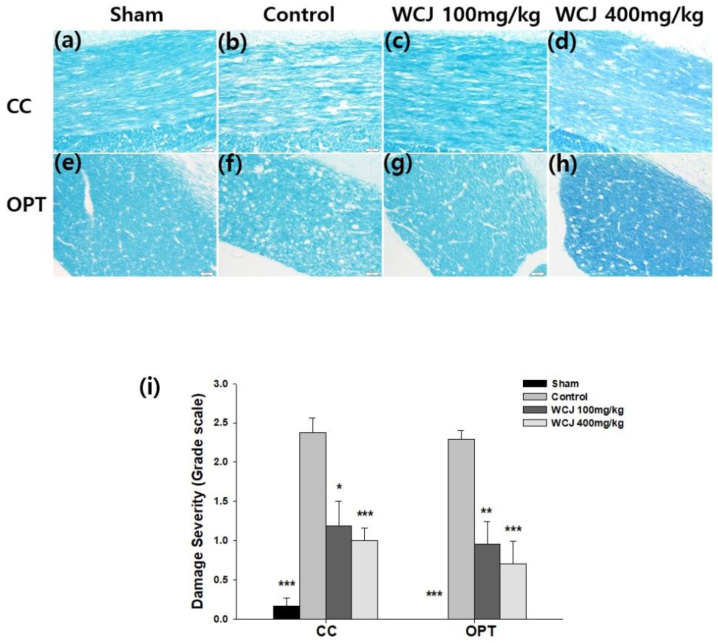
Assessment of white matter damage through WCJ supplementation. (**a**–**h**) Qualitative analysis of white matter damage. Representative photomicrographs of the OPT stained with Luxol fast blue (×200) are presented: (**a**,**e**) sham groups; (**b**,**f**) control groups; (**c**,**g**) WCJ-treated group (100 mg/kg/day); and (**d**,**h**) WCJ-treated group (400 mg/kg/day). (**a**–**d**) and (**e**–**h**) were taken from the CC (corpus callosum) and OPT (optic tract), respectively. (**i**) Quantitative analysis of white matter damage. The severity of damage in the three regions was assessed by an examiner, who was blinded to the experimental conditions and graded the samples as being characterized by normalcy (Grade 0), disarrangement of nerve fibers (Grade 1), formation of marked vacuoles (Grade 2), and disappearance of myelinated fibers (Grade 3). The severity of damage in the CC and OPT is presented using a grade scale. *** *p* < 0.001, ** *p* < 0.01, and * *p* < 0.05 vs. the control group.

Of the white matter regions investigated, including the OPT, CC, and IC, significant damage was only observed in the CC and OPT because they are the two most vulnerable regions in the rat brain [10]. In the qualitative assessment, BCCAO generated white matter rarefaction, which includes vacuolization, in the control group in both the CC and OPT compared with the sham group (Figure 5a–h). On the other hand, white matter rarefaction was reduced in the WCJ-treated groups supplemented with WCJ at dosages of 100 and 400 mg/kg/day in both the CC and OPT compared with the control group. In quantitative analysis, white matter damage in the CC and OPT was graded from zero to three based on the disarrangement and disappearance of nerve fibers consisting of axons and the myelin sheath (Figure 5i) [30]. The scores were significantly reduced (by 50% and 57.9%) in the CC region of the WCJ-treated group supplemented with dosages at 100 and 400 mg/kg/day compared with the control group (*p* < 0.05 and *p* < 0.01, respectively). The scores were significantly reduced as well (by 58.2% and 69.1%) in the OPT region of the WCJ-treated group supplemented with dosages at 100 and 400 mg/kg/day compared with the control group (*p* < 0.01 and *p* < 0.001, respectively). In both cases, the increased dosage of WCJ led to less damage. These findings suggest that WCJ intake over 100 mg/kg/day can inhibit white matter damage in a rat model of chronic cerebral hypoperfusion.

### 3.6. Reduction in Astrocytic Activation through WCJ Supplementation

**Figure 6:**
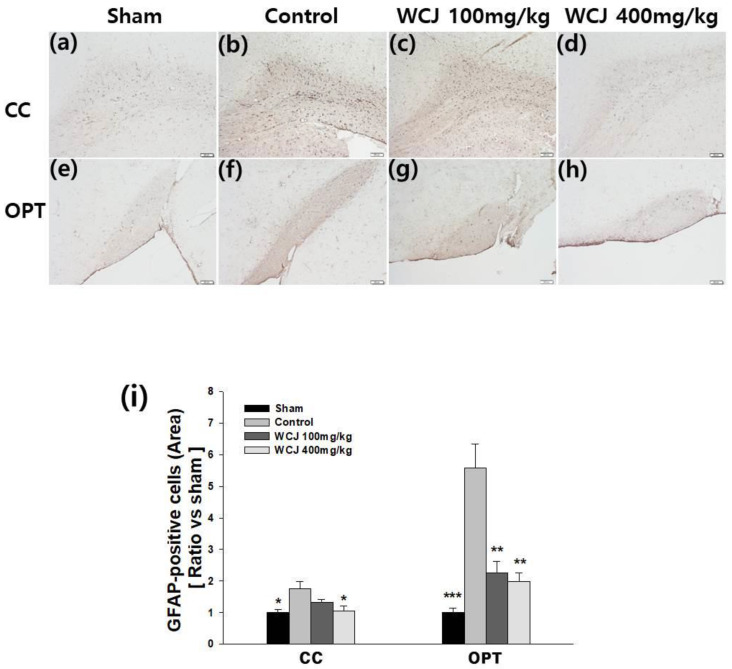
Assessment of astrocytic activation. (**a**–**h**) Representative photomicrographs of astrocytes stained against GFAP (glial fibrillary acidic protein) with immunochemical techniques (H-E, ×100): (**a**,**e**) sham groups; (**b**,**f**) control groups; (**c**,**g**) WCJ-treated group (100 mg/kg/day); and (**d**,**h**) WCJ-treated group (400 mg/kg/day). (**a**–**d**) and (**e**–**h**) were taken from the CC and OPT, respectively. (**i**) Quantitative analysis of GFAP-positive cells. The staining in the CC (corpus callosum) and OPT (optic tract) region was significantly reduced in the WCJ-treated group. *** *p* < 0.001, ** *p*< 0.01, and * *p* < 0.05 vs. the control group.

In the qualitative assessment, BCCAO induced more intense immunohistochemical staining with GFAP in the control group than the sham group in both the CC and OPT (Figure 6a–h). On the other hand, the intensity of the immunohistochemical staining was reduced in the WCJ-treated groups, especially those supplemented with WCJ at a dosage of 400 mg/kg/day. In the quantitative analysis, the intensity of the staining of the samples in the WCJ-treated and control groups was calibrated by setting the sham group’s value as one. The staining in the CC region was significantly reduced by 39.3% in the WCJ-treated group supplemented with WCJ at a dosage of 400 mg/kg/day compared with the control group (*p* < 0.05) (Figure 6i). The staining in the OPT region was significantly reduced by 59.5% and 64.5% in the WCJ-treated group supplemented with WCJ at a dosage of 100 and 400 mg/kg/day, respectively, compared with the control group (*p* < 0.01 and *p* < 0.01, respectively). These findings indicate that WCJ supplementation can inhibit astrocytic activation in the CC and OPT.

### 3.7. Reduction in Microglial Activation through WCJ Supplementation

**Figure 7:**
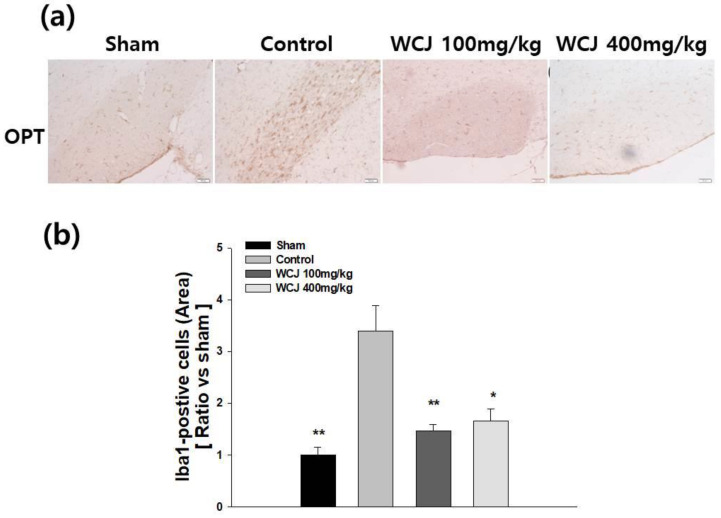
Assessment of microglial activation. (**a**) Representative photomicrographs of microglia stained against Iba1 (ionized calcium-binding adaptor molecule) with immunochemical techniques in the OPT (optic tract) (H-E, ×200). (**b**) Quantitative analysis of Iba1-positive cells. ** *p* < 0.001 and * *p* < 0.05 vs. the control group.

In the qualitative assessment, BCCAO induced more intense immunohistochemical staining with Iba1 in the control group than the sham group in the OPT (Figure 7a). On the other hand, the intensity of the immunohistochemical staining was reduced in the WCJ-treated groups, especially in the one supplemented with WCJ at a dosage of 400 mg/kg/day. In the quantitative analysis, the intensity of the staining of the samples in the WCJ-treated and control groups was calibrated by setting the sham group’s value as one. The staining in the OPT region was significantly reduced by 57% and 51.4% in the WCJ-treated group supplemented with WCJ at dosages of 100 and 400 mg/kg/day, respectively (*p* < 0.01 and *p* < 0.05, respectively) (Figure 7b). These findings show that WCJ supplementation can inhibit microglial activation in the OPT.

## 4. Discussion

In this study, we first determined the optimal ratio between wheat bran, sliced citrus peel, and sliced jujube to prepare WCJ through conducting a search of the literature and cell culture experiments. To determine the optimal ratio, response surface methodology was used, from which the optimal conditions were determined using statistical techniques to minimize the number of experiments required [20]. To further reduce the number of experiments required, we first chose the ratio of wheat bran and sliced jujube through performing a search of the literature [13]. Then, we performed C6 cell culture experiments under hypoxic conditions to find the optimal ratio between sliced citrus peel and sliced jujube. From these experiments, we determined the ratio at which C6 cell survival was improved the most. From these combined approaches, we decided on 6:2:1 as the optimal ratio between wheat bran, sliced citrus peel, and sliced jujube in preparing WCJ for further experiments. For the quality control of WCJ, we identified phytic acid as an indicator component representing WCJ.

We first performed in vitro cell culture experiments to investigate the efficacy of WCJ. In these experiments, WCJ treatment improved C6 cell survival under hypoxic conditions by inhibiting apoptotic cascades, which was assessed through the TUNEL assay and Western blotting for Bcl-2 and Bax. Oligodendrocytes also died by apoptosis in vitro under hypoxic conditions [33,34] and in vivo in BCCAO models [20]. Therefore, our findings suggest that WCJ can protect against white matter injury in the BCCAO model. To test this hypothesis, we performed experiments using a rat BCCAO model. In the experiments, we showed that WCJ supplementation inhibited astrocytic and microglial activation, reduced myelin damage in the CC and OPT, and improved the PLR at dosages over 100 mg/kg/day. Under chronic cerebral hypoperfusion conditions generated in the rat BCCAO model, astrocytes and microglia were activated, leading to white matter damage [4,5,32]. Of the three white matter regions investigated in this study, the OPT, which is involved in the PLR as well as the visual pathway critical to vision-related memory, was damaged [9,12,23]. In addition, the CC, which connects the two cerebral hemispheres, is involved in cognitive status [35], and damage to the CC is associated with VaD [36]. Based on these findings, we concluded that WCJ consumption can prevent VaD because WCJ consumption inhibits white matter damage, which is a hallmark of hypoperfusion dementia and subcortical VaD.

The efficacies of WCJ in treating VaD may derive from the synergistic effects contributed by each ingredient because each ingredient showed efficacies in various conditions related to VaD. For example, WBE supplementation reduced white matter damage in the rat BCCAO model [11], and WBE intake improved vision-related memory in cognitively normal, elderly adults with subjective memory impairments [12]. Citrus peel reduced brain injury in a rat model of ischemic stroke, which is known to cause VaD [18]. Citrus peel intake also reduced the risk of incident dementia [17]. Jujube was revealed to protect against ischemic damage in the gerbil hippocampus via its antioxidant effect [37].

In our previous studies, a 400 mg/kg/day WBE supplementation reduced white matter damage in the rat BCCAO model [11]. Furthermore, in a randomized controlled trial, a WBE intake of 3 g/day improved vision-related memory in cognitively normal, elderly adults with subjective memory impairments [12]. In this study, we showed that a WCJ supplementation of over 100 mg/kg/day reduced white matter damage in the rat BCCAO model, suggesting that WCJ intake might also prevent VaD. It is of note that WCJ has advantages over WBE. Firstly, the large-scale production of WCJ is simpler than that of WBE. Whereas the large-scale production of WCJ only requires standard procedures, such as extraction, filtration, concentration, and drying, the large-scale production of WBE requires additional procedures, including the removal of starch using cold water and centrifugation with a decanter [11]. Secondly, the dosage of WCJ required for efficacy in rats is 100 mg/kg/day, which is equivalent to 1 g/day for a 60-kg person calculated using the conversion factor provided by the US Food and Drug Administration (FDA) [38]. This dosage (1 g/kg) is lower than that of WBE (3 g/kg). Thus, patients with VaD can take three capsules of WCJ per day instead of nine capsules (500 mg unit) of WBE per day (or three capsules of WBE three times a day) [12].

This study has five limitations. First, the main active constituents were not measured, although we identified phytic acid in wheat bran as an indicator component. However, we can predict the main active constituents of each plant. The main active constituents in WCJ are cell wall polysaccharides and phenolic compounds. For example, the main active cell wall polysaccharide of wheat bran is arabinoxylan, which is composed of a xylose backbone with arabinose side chains [39]. We have shown that the administration of arabinoxylan reduced white matter damage and improved memory impairments in the same model of vascular dementia as the one used in this study [40]. The main active phenolic compound of wheat bran is ferulic acid [41]. The administration of ferulic acid reduced oxidative stress and improved the cholinergic system in the hippocampus, as well as improving memory impairment in a rat BCCAO model [42]. The main active cell wall polysaccharide of citrus peel is pectin, which is composed of several backbones with various side chains [43]. The administration of citrus pectin inhibits inflammation and alleviates cerebral stroke, which is also responsible for VaD [44]. We showed that the main active phenolic compounds of citrus peel were nobiletin and tangeretin [18]. The administration of nobiletin activates CaMKII and ERK signaling, as well as improves memory impairment in a short-term mouse BCCAO model [45]. Jujube also contains polysaccharides, which, in terms of weight, make up more than 50% of its composition and exhibit an anti-oxidant effect [46]. The main active phenolic compound of jujube is catechin [47], which protects brain function [19].

Second, we did not show a clear relationship between the in vitro results and the in vivo results. For in vitro results to be relevant to in vivo results, two conditions must be satisfied: the first is that C6, the cell line used in this study, should have oligodendrocyte characteristics; oligodendrocytes are responsible for the myelination of axons [48]. C6 cells express A2B5, which is a cell surface glycolipid used as a biomarker for oligodendrocytes, and galactocerebroside, which is a major glycolipid used as a biomarker for myelin lipid [21]. Therefore, it is suitable to use the C6 cell line, which represents oligodendrocyte. The second condition is that cell wall polysaccharides and phenolic compounds, which are active constituents, should at least be absorbed from the gastrointestinal tract into the bloodstream. Arabinoxylan and pectin, the main active cell wall polysaccharides of wheat bran and citrus peel, respectively, can be taken up into the bloodstream through microfold (M) cells in the Peyer’s patches in the intestine [49,50]. In addition, ferulic acid and nobiletin, the main active phenolic compounds of wheat bran and citrus peel, respectively, can be absorbed directly into the bloodstream [51,52]. Therefore, both the main active cell polysaccharides and phenolic compounds can be absorbed into the bloodstream. In summary, it can be concluded that the in vitro results can be extended to the in vivo results.

Third, we did not include a positive control group. A positive control group should be included in order to evaluate the therapeutic effects of WCJ more accurately. However, there are not yet any FDA-approved drugs used to treat VaD that can be used as a positive control [53]. Therefore, a positive control was omitted in the experiments.

Fourth, behavioral tests were not performed. Our study could be improved by showing that WCJ improves memory impairment using behavioral tests. However, white matter damage is correlated with cognitive impairment in humans [54]. In addition, we have shown, using the Morris water maze test, that the hot water extract of ground wheat improved memory impairment. We have also shown that it reduced white matter damage using the same BCCAO model as the one used in this study [40]. These results suggest that a reduction in white matter damage due to the administration of WCJ could also improve memory impairment. Thus, it might be acceptable to state that a reduction in white matter damage through the administration of WCJ could be effective in treating VaD, the cognitive impairment of which is caused by white matter damage.

Fifth, the neurotransmitters involved in cognition, such as glutamate, were not measured. Although a low level of glutamate can be produced and released from an axon as a neurotransmitter and can bind to the AMPA and NMDA receptors located on myelin in the white matter (so-called axo-myelin synapse) [55], the majority of neurotransmitters are released from presynaptic neurons in the gray matter [56], which is not our region of interest in this study. Based on this knowledge, we did not measure any neurotransmitters in this study.

## 5. Conclusions

We determined 6:2:1 to be the optimal ratio of wheat bran to sliced citrus to sliced jujube in the preparation of WCJ decoction based on information obtained by first searching the literature and then conducting cell survival experiments. We proved that only wheat bran contains phytic acid; consequently, the amount of wheat bran used for the preparation of WCJ can be calculated from the phytic acid content in WCJ. Therefore, phytic acid can be used as an indicator component for quality control. We confirmed the efficacy of WCJ as a plausible herbal medicine for preventing VaD using a rat BCCAO model because the supplementation of WCJ reduced white matter injury and improved the pupillary light reflex through the inhibition of glial activation. As all the ingredients constituting WCJ are edible and an intake of only 1 g/day of WCJ is required for a 60-kg person, WCJ can be developed as a safe medicine or even food to prevent VaD without concerns about toxicity. This is significant considering that there is not yet a proven treatment for VaD approved by the FDA.

## Figures and Tables

**Table 1:** Phytic acid contents in each constituent and WCJ.

	Wheat Bran	WBE	Citrus	Citrus Extract	Jujube	Jujube Extract	WCJ
Content (%) ^a^	3.5 ± 0.17	5.2 ± 0.21	0.059	0.049 ± 0.014	0.051	0.10 ± 0.10	2.2 ± 0.14
Trial numbers	2	3	1	2	1	2	2

WCJ: A decoction containing an optimal ratio of wheat bran, sliced citrus peel, and slice jujube. WBE: wheat bran extract. ^a^ g phytic acid/100 g sample.

**Table 2:** The effect of WCJ supplementation on the pupillary light reflex.

	Sham	Control	WCJ 100 mg/kg	WCJ 400 mg/kg	*p*-Value
Loss of PLR	0 (0%) ^a^	5 (83%)	3 (50%)	1 (17%)	0.015
Normal PLR	6 (100%)	1 (17%)	3 (50%)	5 (83%)	

PLR: pupillary light reflex. The numbers of rats used in the sham, control, and WCJ-treated groups (100 and 400 mg/kg/day) were six, six, and six, respectively. ^a^ Number of rats in each group and percentage (100 × number of rats/number of total rats in each group).

## Data Availability

The datasets used and/or analyzed during the current study are available from the corresponding author upon reasonable request.
